# Medical Opinion and Movement

**Published:** 1909-11-13

**Authors:** 


### Medical Opinion and Movement.

Many and various are the opinions expressed as to the value of the Fulguration method of treatment of Cancer. It is therefore interesting to hear the latest word of Keating-Hart, the author of the treatment, on the subject. At a recent meeting of the *Société de Médecine*, of Paris, he gave the results of his treatment up to date. His report covers a series of 247 cases. The serious cases, considered inoperable by the surgeons, but in which the treatment could be applied thoroughly, have given 63 per cent. of cures, lasting at the most three and a half years, and at the least six months. In operable cases, in which excision of the growth could be practised in combination with the fulguration, there has not as yet been a single failure. The majority of cases consist in cancers of the breast, the bones, and the mucous surfaces—tongue, rectum, etc. The author is convinced that failures with his method at the hands of other operators are entirely due to errors in technique, sometimes surgical, but usually electrical.

Dr. Vigoroux develops the theme in *La Clinique* that Melancholia is connected with some disorder of the sympathetic nervous system. Melancholia is characterised by certain mental and physical symptoms and signs. The mental symptoms consist in a feeling of misery, depression, with slowness of ideation, etc. The physical signs bear a close resemblance, as the author points out, to those of neurasthenia—general feebleness, fatigue at the least effort, headache, palpitation, insomnia. At a more advanced stage in the disease vaso-motor troubles are more pronounced—cold extremities, absence of peripheral pulse, anorexia, constipation, alternating with attacks of serous diarrhœa, diminution of secretion of gastric juice, sweat, and saliva. The author suggests that these symptoms are dependent upon changes in the sympathetic nerves or vaso-motor centres. A case is cited in which melancholic depression developed in a patient suffering from tuberculosis, and at the autopsy the semi-lunar ganglia were found to be affected with a chronic inflammation. He thinks, therefore, that these lesions of the sympathetic were the organic basis underlying the melancholic condition.

At a meeting of the Belgian Academy of Medicine Dr. Weekers, of Liége, gave the results of his researches on the pathology of ocular phlyctenules. With the idea that these phlyctenular lesions are probably of a tubercular nature, he has tested a large number of patients afflicted with this trouble by the cuti-reaction, and obtained a positive result in ninety-one per cent. of the cases. He states further that there was a tuberculous heredity in fifty-six per cent. of the cases. The author suggests that the phlyctenules are produced by toxins circulating in the blood and setting up a reaction at the conjunctiva, and he points to a similarity between them and certain tuberculous affections of the skin. He concludes therefore that these conjunctival lesions may be regarded as an attenuated form of tuberculosis.

Drs. Emile Weil and G. Boyé have made some interesting researches upon the effects of extracts of the anterior and posterior lobes of the pituitary body on the coagulation of the blood in rabbits and in man. With a normal rabbit extracts of the posterior lobe accelerate coagulation. With a rabbit after injection of an extract of leeches, the pituitary extract tends to correct the incoagulability produced by the leech extract, but not invariably so. Extracts of the anterior lobe tend to retard coagulability. In a normal man extracts of the posterior lobe almost always accelerate the coagulability of the blood, and they correct the incoagulability of the blood of a hæmophilic. Extracts of the anterior lobe produce a markedly retarding effect on coagulation, and in hæmophilics render the blood still less coagulable, so that this antagonistic effect of extracts of the two lobes is even more marked in man than in rabbits.

Dr. M. Degny discusses in the form of a treatise the question whether Arsenic should Supplant Mercury in the Treatment of Syphilis. The subject has received a great deal of attention of late among French syphilogists. The way in which preparations of arsenic, notably atoxyl, have come to be regarded as rival medicaments to mercury in the treatment of syphilitic affections is already well known. Atoxyl was found to be a valuable therapeutic agent in trypanosomiases, such as sleeping sickness and frambœsia; then came the discovery of the spirochæta pallida of syphilis and its close relationship to the trypanosomes, and from this the deduction that a drug found efficacious against the one class of micro-organisms, the trypanosomes, might also prove effectual against the closely related spirochæte. Dr. Degny, however, in his critical survey of the question finds no evidence of the superiority of atoxyl over mercury. He maintains that the most obstinate cases will almost invariably yield to mercury by an appropriate method of administration—in one perhaps by inunction, in another by injection, and so on—and cases have yet to be demonstrated in which failure with proper mercurial treatment has been followed by a rapid cure with arsenic. On the contrary, the effects of atoxyl upon most syphilitic manifestations are slower and less certain, and according to this author, tertiary lesions are much less easily affected by atoxyl than by mercury and iodides. But secondary to the relative efficacy of the two drugs arises the question of the relative dangers of the two forms of treatment, and here again Dr. Degny gives the preference to mercury. The well-known toxic symptoms of mercury can generally be successfully combated by appropriate treatment before they assume alarming proportions. But great intolerance is frequently shown to a continued administration of atoxyl. The drug tends to accumulate in the system, and serious symptoms may appear with dramatic suddenness. In addition to the general symptoms there is the danger of amaurosis with optic atrophy, of which several cases have already been reported. In view of these facts the author sees no reason for the substitution of mercury by atoxyl in the treatment of syphilis.

---

The modern conceptions on the subject of Eclampsia are summarised by Dr. R. D. Maxwell in the *London Hospital Gazette* as follows: Eclampsia is only one of the many expressions of toxæmia of pregnancy. The main stress of the toxæmia falls primarily upon the liver, changes in which can always be demonstrated at autopsy. Renal degeneration and albuminuria, though nearly always present, need not necessarily exist. All the toxæmias of pregnancy, from the mildest to the severest, can be explained by a common factor of a fœtal toxin, arising in connection with pathological conditions of the placenta. Such pathological conditions may be explained by undue mixture of the fœto-maternal circulation caused by unusual placental permeability, or by excessive formation of anti-bodies (syncitolysin) in the maternal blood as a defence against its invasion by fœtal cells, syncytium. Human blood reacts constantly to experimental mixtures with placental tissues providing it with specific precipitin properties, and such specific properties will assist in the diagnosis of pregnancy. Lastly, the expectant treatment of eclampsia in the gravest cases of suppression and deep coma is not sound if the origin of the toxæmia in the placenta is admitted; the occurrence of post-partum fits does not necessarily negative this inference, and in the special type of the gravest form of eclampsia *accouchement forcé* is absolutely indicated.

---

In a previous issue (The Hospital, April 3, 1909, p. 6) the curious condition of Avulsion of the Tubercle of the Tibia, known also as Schlatter's disease, was commented upon. A case identical in essentials with those reported at that time was exhibited at a recent meeting of the West London Medico-Chirurgical Society, and led to an interesting discussion. Many members claimed to have had similar cases under observation; this confirms the idea already expressed that the lesion is not so much rare as unreported, and supports also the description of the condition as a definite clinical entity, with, probably, a definite pathological basis. The main symptoms are pain in the region of the insertion of the patellar tendon, especially on severe exertion, such as football playing, and tenderness at the same point. Skiagrams show a solution of continuity of the bone at this point, where normally there is but a single centre of ossification developed. It is therefore possible that on occasions a separate centre of ossification forms, and that the pull of the powerful quadriceps tendon on this sets up an epiphysitis, a periostitis, or even a mild degree of avulsion hardly to be dignified by the term fracture. The lesion is symmetrical as a rule, which favours this hypothesis; it occurs commonly about thirteen to sixteen years of age, and is never seen after twenty, by which time the epiphyses at the head of the tibia are firmly ossified to the shaft. It must be added that two surgeons who took part in the discussion thought that the boy shown was really the subject of a sub-tendinous cyst, and that an exploring syringe or a tenotomy knife should be used in the treatment. The prognosis of Schlatter's disease is so good that treatment is as a rule unnecessary. Painting with iodine, fomentations, and other local applications are said sometimes to be of benefit.

---

The Symptoms and Diagnosis of Pancreatitis are considered by Dr. C. N. Smith in an exhaustive review of the Surgical Aspects of Pancreatitis in the *Annals of Surgery*. At the present day he regards the physical, chemical, and microscopical examination of the patient and his excreta as so complete that it renders the diagnosis of an existing pancreatitis a certainty. The digestive disturbances are too indefinite to be of diagnostic value, though loss of appetite with a particular distaste for meat and fat is very frequent. Vomiting is common in acute pancreatitis, but rare in the chronic form. The fæcal evacuations are frequent, soft, bulky, and pale. Patients often complain of diarrhœa, but the term is usually misapplied, for the stools, though frequent, are large and formed. The importance of this symptom is much increased if jaundice is present, an event commoner in inflammation than in malignant disease of the pancreas. If the ingestion of fats is not diminished the stools may be distinctly greasy; the large size of the latter is chiefly due to the incomplete digestion of albuminous foods, and their frequency to the increased bulk. The normal pigmentation of the fæces is due to the interaction of the pancreatic juice and the bile, and therefore the absence of either will result in unpigmented fæces. The presence in the fæces of undigested muscle fibres is a valuable sign of pancreatic disease, but more so of malignant disease than of inflammation. Steatorrhœa is a more trustworthy sign than this; it can only be determined definitely by thorough chemical examination. Both these last signs may be present without pancreatic lesion if enteritis exists.

---

The existence of pancreatic disease being established, the differential diagnosis of Cancer and Inflammation is of great importance. In cancer of the head of the pancreas, owing to the obstructive gripping of the bile duct by the growth, the fæces will be found free from (or containing but slight traces of) stercobilin. In obstruction of the common duct by stone, or in swelling of the head of the gland due to chronic inflammation, the constriction of the duct is rarely either absolute or continuous, and stercobilin is found in the fæces, though in diminished quantity. Cammidge's reaction is strongly, if not absolutely, confirmatory of the diagnosis of pancreatitis. No one symptom is so reliable as this when considered in connection with the result of examination of the fæces. It is, however, obtained in about 25 per cent. of cases of malignant disease of the pancreas, owing to associated inflammation. Loss of weight is a constant and important symptom both of inflammation and cancer, but is more marked in the latter. Jaundice may be of either biliary or pancreatic origin, for cholecystitis precedes and sets up pancreatitis in the majority of cases of the latter condition. There is a decided tendency to hæmorrhage in the course of pancreatic disease, sometimes important in diagnosis. Pain and tenderness may be pronounced in all forms of pancreatitis, or may be at all times absent. In hæmorrhagic pancreatitis the pain is frequently colicky and paroxysmal. A tumour is rarely to be made out, though in exceptional cases once can be made out. The symptoms of acute hæmorrhagic pancreatitis are, as is well known, practically those of acute peritonitis or, even more often, of intestinal obstruction.

---

Dr. Chamberlen, of Edinburgh, in the time of King Charles II., a celebrated practitioner in midwifery, was the inventor of some very excellent purgative pills, which were, and possibly still may be, sold under the name of Chamberlen's Pills. It may be of interest to note that the following is the authentic direction for their preparation: "Take three pound of best Barbadoes, or Cabolin Aloes (but the Cabolin in preference if it be had), one pound of best Scammony of Aleppo, half-a-pound of Socotrin Aloes. Let all be beaten into a fine powder and put through a search, mixing the same with as much Aqua Apodemia as will make a proper mass. The pills to be smoothed with oil of cloves." The word "Cabolin" presumably signifies "caballin," a coarse variety of aloes used for horses. Aqua Apodemia probably means "hot water," Apodemia being a transcription of "Apo zeme," a decoction.

---

The rate of cooling of several poultice masses has been the subject of a research by Dr. J. D. Pilcher, who has published his results in the *Journal of the American Medical Association* (March 6, 1909). The poultice masses were placed in bottles, a thermometer being supported in each of the latter by a cork. The bulb of the thermometer was adjusted 2.5 cm. from the bottom, and 2.75 cm. from the sides. The various bottles and their contents were heated in a water bath until a uniform temperature of about 95° C. was reached when they were transferred to a second large but shallow water bath, about 2.5 cm. deep, kept at 37° C. to 40° C.—that is to say at about ordinary fever heat. The parts of the bottles above the level of the water were wrapped in cotton. This arrangement was thought to approach as nearly as possible to the conditions of the poultices when applied to the human body. The temperatures were recorded at ten-minute intervals, until the temperature of the bath was reached. The results show that nearly all the poultice masses cool to body temperature in about three hours, or rather less. While the rate of cooling of Cataplasma Kaolini does not differ conspicuously from that of the linseed poultice mass, the difference was constantly in favour of the latter. Dry Kaolin cools appreciably faster than the Cataplasma Kaolini, while glycerine cools considerably more slowly. Other poultice masses that were tried included oatmeal, bread, and corn meal, and these retain their heat even better than the linseed mass; on the other hand, plain water cools at about the same rate as Cataplasma Kaolini, and petrolatum a trifle faster.

Descos and Vial in *La Loire Médicale* call attention to a case of diphtheritic palsy cured by repeated injections of antidiphtheritic serum. Their observation serves to confirm the opinion of Sicard that diphtheritic palsies which show a tendency to become generalised are almost always fatal unless serum-therapy intervenes. The patient, a woman of thirty, was attacked with paralysis of all four limbs. All treatment was of no avail, and the paralysis gradually spread to the circulatory and respiratory organs. Serum treatment was then tried as a last resort, and in the course of five injections during 8 days 110 c.c. of serum were injected. The paralysis was almost immediately overcome and a cure resulted. It may be argued that the paralysis already had a tendency towards spontaneous cure, since the paralysis of the soft palate, tongue, and eyes had passed off without treatment, but nevertheless, the paralysis of the limbs showed no such tendency, the patient having remained immobile for over a month. It is also of importance to remember that a general paralysis of this nature is always a serious matter, and a fatal termination is by no means rare. Out of eleven such cases seen by Haushalter and treated without serum the mortality amounted to 35 per cent. Serum treatment in such cases is somewhat of a novelty, but it is nevertheless a procedure that finds ardent champions in Comby and Netter. According to the conclusions of the former, every patient attacked by diphtheritic palsy, whether recent or of old standing, and whether the symptoms be local or general, should at once be treated by injections of serum which should be repeated every third, fourth, or fifth day, according to the gravity of the case. From 10 to 20 c.c. should be administered on each occasion, and from 60 to 80 c.c. or even more, in all. This treatment is followed by no serious consequences.

---

Although deaths from swallowed chloroform are not very frequent, Liquid Chloroform is sometimes taken in quantity for suicidal purposes. From its general insolubility in water it is practically impossible to remove it from the stomach by washing out with water. Dr. Werth was once called in to a case in which prolonged and copious water-washing of the stomach failed to remove sufficient chloroform to prevent death. He therefore successfully substituted oil for water in a subsequent case, that of a subject who had swallowed 90 gm. of the anæsthetic. After having evacuated the stomach, washings were performed with 2 litres at a time of sesame oil, warmed to 30° C., until all odour of chloroform was removed from the irrigation fluid. This took an hour and a half, and the method had to be suspended on account of imminent asphyxiation. After restoring breathing by means of artificial respiration, the washing was continued. The patient then began to react, and finally to speak. Three hundred mils of blood was then drawn off, followed by subcutaneous injection of two litres of saline solution. Recovery resulted. Not only is oil a good solvent, but it protects the gastric mucous membrane and prevents severe gastritis.

